# Impact of *Hibiscus sabdariffa* Incorporation on Physicochemical Properties, Color Attributes, and Antioxidant Activity of Mead

**DOI:** 10.1111/1750-3841.71439

**Published:** 2026-09-04

**Authors:** Luiz Antônio Teixeira Pedott, Lucas Henrique do Nascimento, Laís Thomazoni, Jamile Zeni, Eunice Valduga

**Affiliations:** ^1^ Department of Food Engineering Universidade Regional Integrada do Alto Uruguai e Missões Erechim Brazil

**Keywords:** anthocyanins, antioxidant activity, flavonoids, Hibiscus sabdariffa, mead, phenolics

## Abstract

The incorporation of botanical ingredients into mead has emerged as a promising strategy for developing fermented beverages enriched with natural bioactive compounds with improved physicochemical quality and bioactive properties. This study evaluated the fermentation performance of mead production and the effects of post‐fermentation incorporation of a hydroalcoholic *Hibiscus sabdariffa* extract on the physicochemical characteristics, color attributes, and antioxidant activity of the final beverage. Two formulations were produced: a control mead and a hibiscus‐enriched mead containing 20% (v/v) extract. Fermentation kinetics demonstrated adequate fermentative performance despite incomplete sugar utilization, resulting in beverages with moderate ethanol content (∼4.7% v/v) and residual sugar concentrations of approximately 37 g/L. The hydroalcoholic extract contained high concentrations of total phenolics (1.59 mg GAE/mL), total flavonoids (0.091 mg EQ/mL), and total anthocyanins (0.441 mg CE/mL), which contributed to an approximately 10‐fold increase in antioxidant activity, increased titratable acidity, a lower pH, reduced luminosity (L*), and higher redness (a*) owing to the presence of anthocyanin pigments and naturally occurring organic acids. Overall, post‐fermentation incorporation of H. sabdariffa enhanced the physicochemical, chromatic, and functional quality of mead while preserving the fermentation characteristics previously achieved, highlighting its potential as a multifunctional natural ingredient for clean‐label, value‐added fermented beverages.

**Practical Applications:**

The incorporation of *H. sabdariffa* into mead improved physicochemical, visual, and functional properties while preserving the alcohol content and residual sugar profile established during fermentation. Hibiscus enhanced acidity, red coloration, and antioxidant activity, highlighting its potential as a natural colorant and source of bioactive compounds in clean‐label fermented beverages. These results support the development of innovative, value‐added mead formulations with greater functional and commercial appeal.

## 1 Introduction

Mead is one of the oldest fermented beverages in the world and has recently gained renewed commercial and scientific interest, driven by the expansion of the craft beverage sector and increasing consumer demand for functional, clean‐label, and naturally derived alcoholic beverages (Essiedu and Kovaleva 2024; Habschied et al. 2025). Produced through the alcoholic fermentation of honey diluted in water, generally by yeasts of the genus *Saccharomyces*, mead represents a versatile fermentation matrix with significant potential for technological innovation and product diversification (Mendes‐Ferreira et al. 2010; Pereira et al. 2009). Its composition, including sugar profile, acidity, volatile compounds, and bioactive constituents, depends on the botanical origin of honey and fermentation conditions, which directly influence the physicochemical, sensory, and functional characteristics of the final product (Webster et al. 2025; García et al. 2024; Deng et al. 2024; Cuenca et al. 2022; Kawa‐Rygielska et al. 2019). Driven by the growing demand for clean‐label and functional beverages, the incorporation of plant‐derived ingredients has become an important strategy to improve both technological and nutritional quality (Hidalgo‐Fuentes et al. 2024; Yuan et al. 2021).

Among these ingredients, *Hibiscus sabdariffa* has attracted considerable attention because it is naturally rich in anthocyanins, flavonoids, phenolic compounds, and organic acids, which are associated with antioxidant, anti‐inflammatory, and antihypertensive activities (Da‐Costa‐Rocha et al. 2014; Ojulari et al. 2019). From a technological perspective, hibiscus anthocyanins, particularly cyanidin‐3‐sambubioside and delphinidin derivatives, provide intense red coloration and exhibit good stability under acidic conditions, making hibiscus a promising natural colorant for fermented beverages (Shruthi and Ramachandra 2019; Da‐Costa‐Rocha et al. 2014). In addition, the presence of phenolic compounds and organic acids may enhance antioxidant capacity, modify acidity, and improve product stability and sensory appeal (Da‐Costa‐Rocha et al. 2014; Vega‐Maturino et al. 2026).

Previous studies have shown that hibiscus supplementation can modify pH, titratable acidity, color, phenolic composition, and antioxidant activity in fermented beverages (Adamenko et al. 2018; Cicha‐Wojciechowicz et al. 2025; Kawa‐Rygielska et al. 2019). However, its effects on mead fermentation, particularly regarding fermentation kinetics, yeast performance, physicochemical characteristics, color development, and antioxidant properties, remain poorly understood. Although hibiscus has been successfully incorporated into other fermented beverages, studies investigating its influence on the fermentation product and quality attributes of mead are still scarce, representing an important knowledge gap in this field. Therefore, this study aimed to characterize the fermentation kinetics of mead production and evaluate the effects of post‐fermentation incorporation of *H. sabdariffa* extract on physicochemical composition, color attributes, and antioxidant activity.

Furthermore, a better understanding of the interaction between hibiscus bioactive compounds and mead fermentation may support the development of naturally enriched fermented beverages with enhanced technological, functional, and sensory properties. These findings may also provide a scientific basis for future studies focused on optimizing hibiscus incorporation levels, evaluating sensory acceptance and storage stability, and expanding the industrial application of botanical ingredients in mead production.

Therefore, this study aimed to characterize the fermentation kinetics and stoichiometric parameters of the control mead during alcoholic fermentation and to evaluate the effects of post‐fermentation incorporation of a *H. sabdariffa* hydroalcoholic extract on the physicochemical, chromatic, and functional properties of the final beverage.

## 2 Materials and Methods

### 2.1 Sample Collection and Preparation

The honey sample, obtained from African honeybees (*Apis mellifera scutellata*) and predominantly from orange blossom, used in this study was collected in September 2022 from a rural producer in Rio Grande do Sul, Brazil (27°41ʹ06ʺS, 52°16ʹ04ʺW). According to the supplier, the honey was predominantly derived from orange blossom nectar; however, no melissopalynological analysis was performed in the present study to independently confirm its botanical origin. The honey was characterized in terms of moisture content, total acidity, hydroxymethylfurfural (HMF) content, and total soluble solids.

Dried hibiscus flowers (10% moisture), belonging to the species *H. sabdariffa*, were purchased from a local market. The hibiscus extract was prepared using an alcoholic solution of mead (control formulation, F1), in which 100 g of dried flowers per liter of mead were added and infused for 10 days in the dark at 22°C ± 2°C. Subsequently, the extract was filtered using Whatman No. 1 filter paper and stored under refrigeration (4°C ± 2°C) in a dark glass bottle, flushed with inert gas (Nitrogen). The extract was filtered through Whatman No. 1 filter paper to remove coarse suspended particles and residual floral material, thereby improving extract homogeneity and preventing the incorporation of insoluble solids into the mead. This filtration method was selected because it effectively clarifies the extract while allowing soluble compounds, including low‐molecular‐weight phenolics and anthocyanins, to remain in solution.

### 2.2 Characterization of the Hibiscus Extract

The hibiscus extract was characterized in terms of pH, total acidity, total phenolic content, total flavonoids, total anthocyanins, and antioxidant activity.

### 2.3 Preparation of Mead Formulations

The methodology employed for mead production (Formulation 1 [F1]) was adapted from Rocha Da Silva et al. (2023). Initially, the must was prepared at a ratio of 1:3 (w:v), resulting in 24 L of must adjusted to 16 °Brix. Immediately after preparation, potassium metabisulfite (8 g/hL) was added to suppress undesirable microorganisms naturally present in the honey and must, minimize oxidative reactions, and provide favorable microbiological conditions for the subsequent inoculation of the selected yeast strain. The sulfited must was then allowed to stand for 12 h before inoculation to ensure adequate antimicrobial action while allowing the free sulfur dioxide concentration to decrease to levels compatible with yeast growth.

The inoculum was prepared using a mixture of 5% (w/v) honey in deionized water and 6 g of Saccharomyces cerevisiae ICV 254 yeast (Lalvin). The yeast was initially rehydrated in accordance with the manufacturer's recommendations at 37°C ± 1°C for 20 min, followed by adaptation to the fermentation medium through successive transfers using the honey solution, corresponding to a final inoculum volume equivalent to 5% of the total must volume. The activated inoculum was then transferred to a 25‐L glass fermenter containing the prepared must. All glassware, fermentation vessels, sampling materials, and accessories used during the fermentation process were previously sanitized and sterilized to minimize the risk of microbial contamination. The fermenter was sealed with a sterilized stopper fitted with an airlock containing 70% (v/v) ethanol to maintain aseptic conditions, prevent external microbial entry, and allow the release of carbon dioxide produced during fermentation.

Alcoholic fermentation was conducted in a refrigerated chamber (Refrigeração Menoncin Indústria e Comércio LTDA, Brazil) at 18°C ± 2°C. During the first 4 days of fermentation, fermentation nutrient—Activit (PERDOMINI‐IOC, Italy)—was added at concentrations of 10, 20, 15, and 15 g/hL on Days 1, 2, 3, and 4, respectively, accompanied by successive delestage operations to improve oxygen availability during the initial stages of yeast growth and promote homogeneous fermentation. The fermentation process was monitored for 254 h through periodic measurements of fermentation parameters, including sugar consumption and ethanol production. The fermentation duration was defined based on the stabilization of reducing sugar concentration and the completion of the main alcoholic fermentation phase.

During the kinetic monitoring period, samples were collected aseptically at predetermined intervals to evaluate fermentation performance and physicochemical changes throughout the process.

At the end of alcoholic fermentation (after 254 h of fermentation), racking was performed, followed by the addition of 10 g/hL potassium metabisulfite to protect the mead against oxidation and microbial spoilage during maturation and storage. The vessel was then flushed with inert nitrogen gas, and colloidal stabilization was carried out in a cold chamber (Refrigeração Menoncin Indústria e Comércio LTDA, Brazil) at 7°C for approximately 40 days, followed by a second racking and bottling under controlled aeration using inert gas. This formulation, prepared without hibiscus extract, was designated as the control treatment (Formulation 1 [F1]).

For the hibiscus mead formulation (Formulation 2 [F2]), a hydroalcoholic *H. sabdariffa* extract was incorporated at 20% (v/v) after fermentation and colloidal stabilization, while all remaining fermentation and maturation conditions were identical to those of the control formulation. This strategy was adopted to preserve anthocyanins and other phenolic compounds by minimizing their loss during clarification, thereby maximizing their contribution to the physicochemical, color, and antioxidant properties of the final mead.

### 2.4 Characterization of Mead Formulations

The mead formulations (F1 and F2) were characterized in terms of alcohol content, pH, total acidity, color parameters (L*, a*, b*, and chroma C*), total phenolic content, total flavonoids, total anthocyanins, and antioxidant activity (2,2‐diphenyl‐1‐picrylhydrazyl [DPPH] radical and ABTS^•+^ radical).

### 2.5 Fermentation Kinetics and Evaluation of Stoichiometric Parameters

In order to obtain stoichiometric parameters and evaluate the kinetics of substrate consumption (total reducing sugars, TRS), cell growth, and ethanol production, periodic samples (every 6 or 12 h) were collected from the fermentation broth of formulation F1.

#### 2.5.1 Global Instantaneous and Specific Rates

From the profiles of global cell concentration, product formation, and substrate (TRS) consumption over time, it was possible to determine, through mass balances for each component, the microbial growth rate (*r*_x_), product formation rate (*r*_p_), and substrate consumption rate (*r*_s_), as described in Equations (1) to (3) (Bailey and Ollis 1986):

(1) $$r_{x}=\frac{dX}{dt},$$

(2) $$r_{p}=\frac{dP}{dt},$$

(3) $$r_{g}=-\frac{dG}{dt}.$$

By dividing the instantaneous rates by the cell concentration at that moment, the specific growth rate (*μ_x_*), expressed in h^−1^, was obtained (Equation 4) (Bailey and Ollis 1986):

(4) $$\mu_{x}=\frac{r_{x}}{X}.$$

For constant rates (exponential phase), these were determined from the slope of the fitted straight line to the curves representing cell growth, TRS consumption, and ethanol production kinetics.

#### 2.5.2 Global Conversion Factors

The global conversion factors of TRS (glucose) into ethanol (product), *Y*_P/G_ (g_ethanol_/g_TRS)_, TRS into cells, *Y*_X/G_ (g_cells_/g_TRS_), and cells into ethanol, *Y*_P/X_ (g_ethanol_/g_cells_), are expressed by Equations (5) to (7):

(5) $$Y_{P/G}=\frac{r_{p}}{r_{g}}=-\frac{dP}{dG},$$

(6) $$Y_{X/G}=\frac{r_{x}}{r_{g}}=\frac{dX}{dG},$$

(7) $$Y_{P/X}=\frac{r_{p}}{r_{x}}=\frac{dP}{dX},$$

where *r*_x_ = cell growth rate (g/L·h); *r*_s_ = TRS consumption rate (g/L·h); *r_p_* = ethanol formation rate (g/L·h).

Biomass and product productivity, specific microbial growth rate, and doubling time are expressed by Equations (8) to (11), respectively.

(8) $$P_{X}=\frac{X_{f}-X_{0}}{t},$$

(9) $$P_{P}=\frac{P_{f}-P_{0}}{t_{\mathrm{fp}}},$$

(10) $$X_{t}=X_{i}\times e^{\mu_{\max}\;\left(t_{1}-t_{0}\right)},$$

(11) $$t_{g}\left(h\right)=\frac{0,693}{\mu_{\max}},$$

where *X*_f_ is the final cell concentration (g/L); *X*_0_ is the initial cell concentration (g/L); *t* is the fermentation time; *P*_f_ is the final product concentration (g/L); *P*_0_ is the initial product concentration (g/L); *t*_fp_ is the final time of product formation (h); e is the exponential function; *µ*_max_ is the maximum specific growth rate; *t*_1_ is the end of the log phase (h); *t*_0_ is the beginning of the log phase (h); *X*_t_ is the cell concentration at the end of the log phase (g/L); and *X*_i_ is the cell concentration at the beginning of the log phase (g/L).

### 2.6 Analytical Determinations

#### 2.6.1 Total Acidity

Total acidity of honey and mead was determined according to the method described by the Instituto Adolfo Lutz (IAL 2008), with modifications. For honey, 2 g of sample was weighed into a 250‐mL Erlenmeyer flask and diluted with 50 mL of distilled water. The solution was homogenized, and two drops of 1% phenolphthalein indicator were added. Titration was carried out with 0.01 mol/L NaOH until a persistent pale pink color was observed, corresponding to pH 8.2–8.4.

For mead, 50 mL of sample was transferred to a 250‐mL Erlenmeyer flask, and titration was performed using 0.1 mol/L NaOH under continuous stirring, with pH monitoring using a previously calibrated pH meter. The endpoint was defined at pH 8.2–8.4. Results were expressed as titratable acidity (mEq/L).

#### 2.6.2 pH

pH was measured using a Tec‐7 pH meter (Tecnal, Brazil), previously calibrated with standard buffer solutions at pH 4.0 and 7.0 at 20°C–25°C. Measurements were performed by immersing the electrode directly in the sample, and values were recorded after stabilization. The electrode was rinsed with distilled water between calibrations and sample readings.

#### 2.6.3 Determination of Total Phenolic Content

Total phenolic content was determined using a methodology adapted from Singleton et al. (1999). Aliquots of 0.5 mL of the sample (diluted 1:100), 2.5 mL of Folin–Ciocalteu reagent (diluted 1:10 in distilled water), and 2 mL of an aqueous solution of anhydrous sodium carbonate (4% w/v) were added and mixed using a vortex mixer (IKA model MS 3B). The solutions were briefly mixed and allowed to stand for 2 h at room temperature, protected from light. Absorbance readings were performed using a UV–Vis spectrophotometer (Logen Scientific, LS‐7052‐BIV) at 760 nm. Results were expressed as gallic acid equivalents per milligram of sample, based on a calibration curve constructed using a commercial gallic acid standard (Neon) at concentrations ranging from 1 to 100 µg/mL.

#### 2.6.4 Determination of Total Flavonoids

Flavonoid content was quantified according to the method described by Garrido et al. (2013), with modifications. Test tubes were prepared by adding 0.5 mL of the sample (diluted 1:10), 4.3 mL of 70% ethanol, 0.1 mL of 10% (w/v) aluminum nitrate, and 0.1 mL of 10% (w/v) potassium acetate. After mixing, the tubes were kept at room temperature, protected from light, for 40 min. Absorbance was measured using a UV–Vis spectrophotometer (Logen Scientific, LS‐7052‐BIV) at 415 nm. Results were obtained via linear regression based on a standard curve constructed using aqueous quercetin solutions (1–100 µg/mL). Distilled water was used as a blank in place of the sample. Data were expressed as milligrams of quercetin equivalents per gram of extract (mg QE/g).

#### 2.6.5 Determination of Total Anthocyanins

The total anthocyanin content was determined according to the method described by da Rosa Fetter et al. (2010). Hibiscus extract was diluted 1:100 in acidified ethanol (pH 2.0) and adjusted with 5 M hydrochloric acid (Química Moderna), and the absorbance was measured at 535 nm using a spectrophotometer. Quantification was performed by interpolation from a calibration curve prepared with cyanidin‐3‐glucoside (Sigma–Aldrich) at concentrations ranging from 1 to 25 ppm, and the results were expressed as milligrams of cyanidin‐3‐glucoside equivalents per milliliter (mg CE/mL). For the blank, acidified ethanol was used in place of both the sample and the standard solution. All measurements were carried out in triplicate.

#### 2.6.6 Moisture

Honey moisture content was determined by an indirect method based on refractive index measurements at 20°C, using the Chataway table (IAL 2008).

#### 2.6.7 HMF

HMF content was determined according to IAL (2008). Briefly, 5 g of sample was mixed with 25 mL of distilled water and transferred to a 50‐mL volumetric flask. Subsequently, 0.5 mL of Carrez I solution (15 g K_4_Fe(CN)_6_·3H_2_O/100 mL) and 0.5 mL of Carrez II solution (30 g Zn(OAc)_2_·2H_2_O/100 mL) were added. The volume was made up to 50 mL with distilled water, and the solution was filtered through Whatman No. 1 filter paper, discarding the first 10 mL.

Aliquots of 5 mL of filtrate were transferred into two test tubes. To the first tube, 5 mL of distilled water was added (sample), while 5 mL of sodium metabisulfite solution (0.10 g NaHSO_3_/100 mL) was added to the second tube (IAL, 2008). Absorbance was measured at 284 and 336 nm using a UV–Vis spectrophotometer.

#### 2.6.8 Total Soluble Solids (°Brix)

Total soluble solids were determined at 20°C using a bench Abbe refractometer (BioBrix).

#### 2.6.9 TRS

Reducing sugars (glucose and fructose) were quantified using the 3,5‐dinitrosalicylic acid (DNS) method described by Miller (1959). Standard glucose solutions (0.1–1.0 g/L) were used to construct the calibration curve. Prior to analysis, samples were centrifuged at 9000 rpm for 5 min to remove yeast biomass. An aliquot (1 mL) of supernatant was diluted to 10 mL with distilled water, and 1 mL of the diluted sample was mixed with 1 mL of DNS reagent (the blank contained distilled water instead of sample). The reaction mixture was heated in a boiling water bath for 8 min, rapidly cooled in an ice bath for 8 min, diluted with 16 mL of sodium potassium tartrate solution, and the absorbance was measured at 540 nm.

#### 2.6.10 Cell Concentration

Cell concentration was estimated by spectrophotometric measurement at 600 nm, based on a calibration curve correlating absorbance and yeast concentration, adapted from Rocha Da Silva et al. (2023). Standard suspensions of commercial yeast (1–35 g/L) were prepared in distilled water to construct the calibration curve. During fermentation, 1‐mL samples were collected at defined time intervals and analyzed at 600 nm against a blank (fermentation medium prior to inoculation).

#### 2.6.11 Alcohol Content

Alcohol content was determined using a Dujardin–Salleron ebulliometer with an Ebulliometer 3300 (Metalúrgica Leonardo Ltda, Brazil) (IAL 2008).

#### 2.6.12 Antioxidant Activity

##### 2.6.12.1 DPPH• Radical Method

Antioxidant activity was determined using the DPPH^•^ radical scavenging method (Brand‐Williams et al. 1995). Briefly, 1 mL of sample (at various dilutions ranging from the undiluted sample to 2.5 × 10^−5^ mL of sample per milliliter of aqueous solution) was mixed with 1 mL of ethanolic DPPH^•^ solution (0.1 mM), vortexed, and incubated in the dark for 30 min at room temperature. Absorbance was measured at 515 nm using a UV–Vis spectrophotometer (Logen Scientific, LS‐7052‐BIV).

The control consisted of 1 mL of ethanol (50%) and 1 mL of DPPH^•^ solution. Sample blanks were prepared to correct for color interference by mixing 1 mL of sample with 1 mL of ethanol. Antioxidant activity (AA%) was calculated according to Equation (12):

(12) $$\mathrm{AA}\%=100-\frac{\left[\left(\mathrm{Abs}.\;\mathrm{sample}-\mathrm{Abs}.\;\mathrm{blank}\right)\times 100\right]}{\mathrm{Abs}.\;\mathrm{control}}.$$

##### 2.6.12.2 ABTS^•+^ Radical Method

Antioxidant activity against the ABTS^•+^ radical was determined according to the method described by Larrauri et al. (1997), with modifications. Initially, 1000 µL of a 7‐mM aqueous ABTS solution (Sigma–Aldrich) was mixed with 17.6 µL of a 140‐mM aqueous potassium persulfate solution (Synth) to prepare the ABTS^•+^ radical solution. This mixture was kept in the dark for 16 h. Afterward, the solution was diluted with ethanol until an absorbance of 0.700 ± 0.05 was reached at 734 nm.

Next, 20 µL of sample solutions (same dilution used for the DPPH^•^ method) were reacted with 2.0 mL of the ABTS^•+^ solution. The mixture was vortexed and kept in the dark for 6 min, and then the absorbance was measured at 734 nm using a spectrophotometer (Logen Scientific, LS‐7052‐BIV). Distilled water was used as the blank, and the control was the ABTS^•+^ solution with 20 µL of distilled water. A calibration curve was also prepared using Trolox (Sigma–Aldrich), with concentrations ranging from 0.0025 to 0.025 mg/mL. Results were expressed as milligrams of Trolox equivalent per gram of sample based on the calibration curve.

#### 2.6.13 Color Parameters (L*, a*, b*, and Chroma C*)

Color parameters (L*, a*, b*, and chroma C*) were determined using a colorimeter (Minolta Chroma Meter CR‐400, Konica Minolta, Japan) operating in the CIELAB color space. The instrument was calibrated before analysis using the manufacturer's standard white calibration plate and the D65 standard illuminant. Measurements were performed using 25 mL of sample in a cuvette with a 2.5‐cm optical path, under 45° illumination.

### 2.7 Statistical Analysis

The results (*n* = 3) of the physicochemical analyses of the beverage samples were statistically analyzed using analysis of variance (ANOVA), and mean comparisons were performed using the Student's *t*‐test with the aid of STATISTICA version 5.0 (StatSoft, Inc. 2012), at a 95% confidence level. Analytical curves, such as those used for determining reducing sugars and antioxidant activity, were constructed by linear regression using Microsoft Excel. The coefficient of determination (*R* ^2^) was used to evaluate the goodness of fit of the models to the calibration curves.

## 3 Results and Discussion

### 3.1 Physicochemical Characterization of Honey and Its Implications for Mead Fermentation

Table 1 presents the physicochemical characteristics of the honey sample used for mead formulation, which are fundamental parameters for raw material quality and are directly related to fermentative performance, microbiological stability, and the technological properties of the final product.

**TABLE 1:** Physicochemical characteristics of honey.

Characteristics	Values
Total acidity (mEq/kg)	32.13 ± 1.61
Moisture (%)	16.27 ± 0.81
Hydroxymethylfurfural (HMF) (mg/kg)	57.06 ± 2.65
Total soluble solids (°Brix)	81.80 ± 0.91

*Source*: Brazil (2000).

The honey acidity showed a value of 32.13 mEq/kg (Table 1), remaining below the maximum limit of 50 mEq/kg established by Brazilian legislation (Brazil 2012). Values within this range are indicative of good quality, since acidity is associated with the presence of organic acids naturally occurring in honey and may reflect both botanical origin and possible fermentative processes (Tôrres et al. 2021). In addition, organic acids contribute to flavor development, microbiological stability, and buffering capacity of the must during fermentation. The observed value is compatible with those reported for Apis mellifera honeys (16.9–49.2 mEq/kg) (Welke et al. 2008), indicating adequate conservation and maturation conditions of the sample.

The moisture content was 16.27%, also in accordance with the maximum limit of 20% established by current legislation (Brazil 2012). This parameter is directly associated with microbiological stability and shelf life of honey, since low water contents reduce water activity and limit the growth of spoilage microorganisms. Honeys with low moisture are considered more stable during storage and less susceptible to spontaneous fermentation. Furthermore, low moisture levels favor higher concentrations of fermentable sugars in the must, an important condition for the production of fermented alcoholic beverages.

The HMF content was 57.06 mg/kg, a value close to the maximum limit of 60 mg/kg established by Brazilian legislation (Brazil 2012). HMF is widely used as an indicator of honey quality and freshness and is mainly formed through the thermal degradation of hexoses during heating and/or prolonged storage (Tôrres et al. 2021). Elevated HMF values may indicate exposure of honey to inadequate processing or storage conditions, promoting sugar degradation and changes in the sensory and technological properties of the product (Shapla et al. 2018).

Although HMF has been reported to inhibit the growth and fermentative activity of *S. cerevisiae* at concentrations substantially higher than those detected in the present study, the HMF content of the honey (57.06 mg/kg) remained within the maximum limit established by Brazilian legislation and is representative of honeys commonly used for mead production. Therefore, HMF is unlikely to have significantly affected yeast metabolism or fermentation performance under the conditions evaluated. Instead, the incomplete fermentation observed is more plausibly attributed to the intrinsic nutritional limitations of honey must, particularly the low availability of yeast assimilable nitrogen (YAN) and essential micronutrients.

The total soluble solids analyses indicated an average value of 81.8 °Brix, reflecting the high sugar concentration characteristic of mature honeys and being directly related to their microbiological stability and fermentative potential. The main sugars present in honey, especially glucose and fructose, constitute important substrates for alcoholic fermentation, favoring ethanol production by yeasts of the genus Saccharomyces (P. M. da Silva et al. 2016; Pereira et al. 2009). However, high sugar concentrations also contribute to increased osmotic pressure in the medium, a condition that may impair cellular adaptation and compromise fermentation kinetics if the must is not properly diluted and nutritionally supplemented (Pereira et al. 2009; Pereira et al. 2015). This effect is particularly relevant in mead fermentations, in which the high soluble solids content may induce osmotic stress and reduce yeast metabolic activity.

Overall, the physicochemical characteristics of the honey indicate suitable raw material for mead production, presenting parameters compatible with quality standards established by legislation and the literature. However, the high sugar content and the HMF level close to the legal limit suggest that factors related to storage conditions and honey composition may influence yeast metabolism and fermentative performance throughout beverage production.

### 3.2 Fermentative Performance and Kinetic Analysis of Mead Production

**FIGURE 1:**
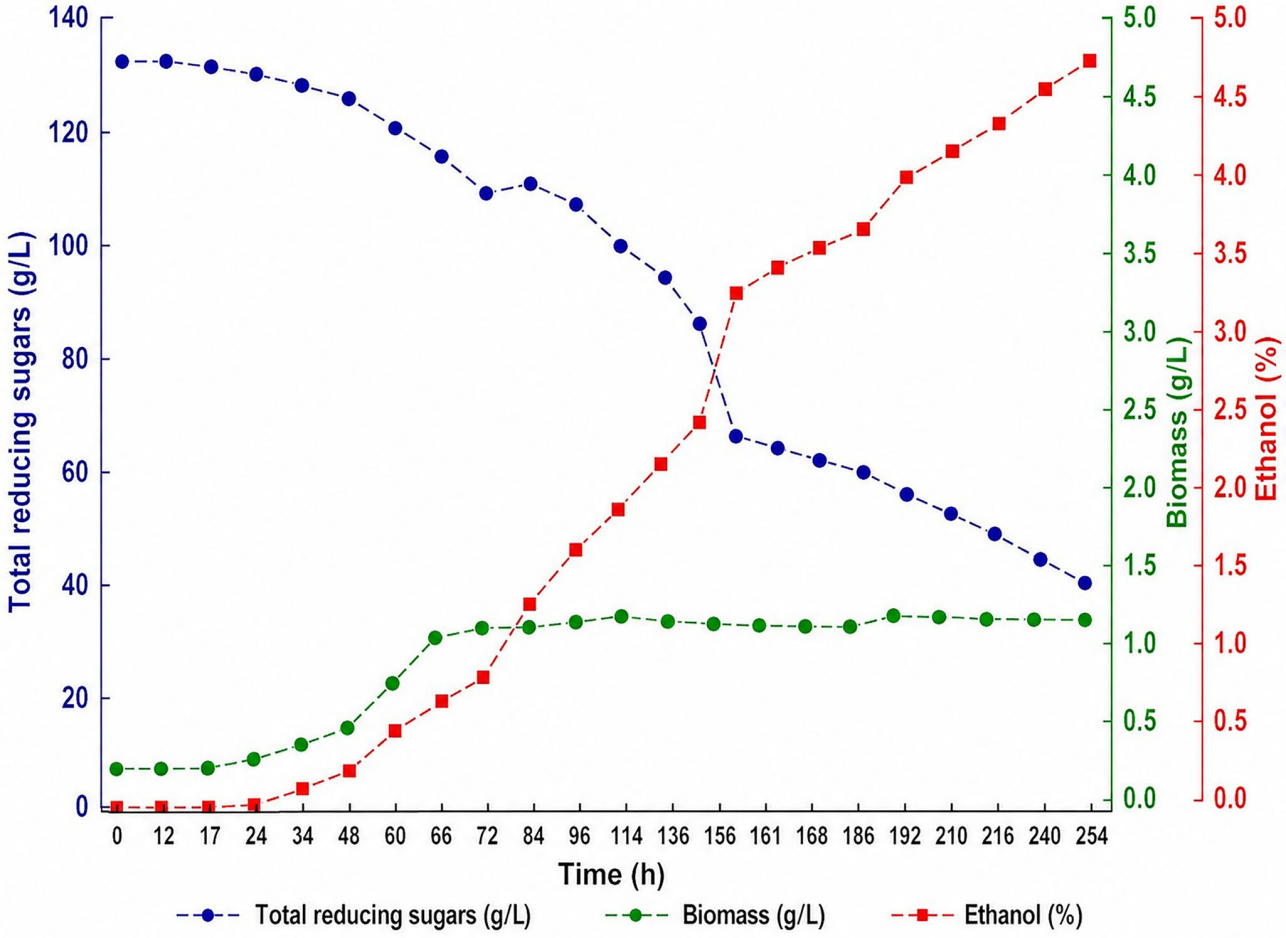
Fermentation kinetics of mead during alcoholic fermentation, showing cell concentration, total reducing sugar consumption, and ethanol production over time.

The fermentation kinetics of mead (Figure 1) showed an initial increase in cell concentration from approximately 0.3 to 1.25 g/L during the first 72 h, followed by biomass stabilization until the end of the fermentative process (294 h). This behavior is consistent with the classical microbial growth model, characterized by lag (0–24 h), exponential (24–72 h), and stationary phases (after 72 h). The interruption of cell growth, even in the presence of residual sugars, suggests nutritional limitations inherent to the honey must, together with the inhibitory effects of accumulated ethanol, factors frequently reported in mead fermentations and other sugar‐rich musts (Pereira et al. 2009; Mendes‐Ferreira et al. 2009).

Honey naturally contains low concentrations of assimilable nitrogen and essential micronutrients required for yeast metabolism, conditions that directly compromise specific growth rate, cell viability, and fermentation efficiency (Mendes‐Ferreira et al. 2009; Bely et al. 1990).

Recent studies have further demonstrated that YAN availability directly modulates the fermentative metabolism of S. cerevisiae, influencing substrate assimilation, fermentation rate, and cellular tolerance to metabolic stress (García‐Ríos et al. 2024). Although YAN was not determined in the present study, the observed fermentation profile is consistent with the compositional characteristics of honey must reported in the literature. Future studies should include the determination of YAN and micronutrient composition to better elucidate their influence on fermentation kinetics and ethanol production.

Simultaneously, a gradual reduction in TRS was observed, from 131 to 37 g/L, indicating progressive substrate utilization for both cell growth and ethanol formation. However, the persistence of residual reducing sugars at the end of fermentation indicates that substrate utilization was incomplete. Therefore, although the substrate‐to‐product conversion coefficient (*Y*_P/S_) remained close to the theoretical value, the overall fermentative performance should be interpreted as moderate rather than highly efficient, most likely because of the intrinsic nutritional limitations of honey must. The slowdown in sugar consumption during the final stages was associated with reduced cellular metabolic activity caused by osmotic stress, ethanol accumulation, and possible loss of yeast viability, phenomena widely described in high‐sugar alcoholic fermentations. Walker and Stewart (2016) reported that high sugar and ethanol concentrations impair plasma membrane integrity, nutrient transport, and cellular homeostasis, progressively reducing the metabolic activity of *S. cerevisiae*. Similar observations have also been reported by G. P. da Silva et al. (2009) and Walker and Stewart (2016).

Additionally, fermentative stress conditions, including high osmotic pressure, ethanol accumulation, and low pH, may induce structural and physiological alterations in yeast cells, impairing viability and fermentation performance (Florez et al. 2024). Recent studies have also demonstrated that previous adaptation of yeast strains to high‐gravity media can minimize osmotic stress effects and improve fermentation performance in sugar‐rich systems (Sica et al. 2025).

Importantly, the fermentation profile observed in the present study reflects the intrinsic metabolic behavior of the honey must, since *H. sabdariffa* extract was incorporated only after completion of alcoholic fermentation. Consequently, the kinetic parameters obtained represent the fermentative performance of the control mead and provide an appropriate baseline for evaluating the subsequent effects of hibiscus on beverage quality. This experimental approach also explains why no differences in alcohol content or residual sugar were observed between formulations (Table 3), confirming that hibiscus supplementation did not interfere with yeast fermentative metabolism. Similar observations have been reported for fermented beverages enriched with botanical extracts after fermentation, where improvements in antioxidant properties and color occur without affecting ethanol production or sugar conversion (Essiedu and Kovaleva 2024; Cicha‐Wojciechowicz et al. 2025).

Moreover, the presence of approximately 37 g/L of residual sugars at the end of the process suggests incomplete or sluggish fermentation, indicating that physiological and nutritional factors limited complete substrate assimilation by the yeasts (Bisson 1999). This metabolic pattern is commonly associated with the intrinsic nutritional limitations of honey must. Honey is naturally deficient not only in YAN but also in essential micronutrients, including vitamins (particularly biotin, thiamine, and pantothenic acid) and minerals such as magnesium, potassium, and phosphate, which are required for optimal yeast metabolism. These nutrients play fundamental roles in enzymatic activity, membrane integrity, nitrogen assimilation, and cellular stress tolerance. Consequently, their limited availability, together with the accumulation of ethanol and other metabolic byproducts during fermentation, may reduce sugar uptake, impair ethanol productivity, and contribute to sluggish or incomplete fermentations, even when fermentable sugars remain available. Although the present study did not determine YAN or micronutrient concentrations, the observed fermentation profile is consistent with the nutritional limitations typically described for honey‐based fermentations (Mendes‐Ferreira et al. 2010; Pereira et al. 2015; García‐Ríos et al. 2024; De Gino Soares et al. 2025).

Collectively, these findings indicate that the fermentative process achieved efficient substrate conversion into ethanol, although with moderate alcoholic yield, possibly limited by the nutritional characteristics inherent to honey must. The relatively slow fermentation observed is also characteristic of honey‐based substrates because their carbohydrate composition differs markedly from grape must. Honey contains predominantly fructose and glucose but is naturally deficient in amino acids, vitamins, minerals, and sterols required for optimal yeast growth. Under these conditions, *S. cerevisiae* redirects part of its metabolic resources toward cellular maintenance and stress adaptation rather than biomass formation, resulting in prolonged fermentation times and incomplete sugar utilization. Moreover, progressive ethanol accumulation enhances membrane fluidization, increases intracellular reactive oxygen species, and reduces ATP availability for active nutrient transport, thereby reinforcing the decline in fermentative activity during the stationary phase (Walker and Stewart 2016; Gibson et al. 2007; García‐Ríos et al. 2024).

These findings reinforce the importance of nutritional supplementation, especially with assimilable nitrogen sources, as a strategy to optimize fermentative performance, increase cell growth rate, and reduce processing time in mead production systems (Bely et al. 1990; Pereira et al. 2015). Recent reviews have demonstrated that moderate nitrogen supplementation in mead and fruit wine fermentations significantly improves cell growth, reduces residual sugars, and increases ethanol productivity, whereas low nitrogen availability is strongly associated with sluggish or incomplete fermentations (De Gino Soares et al. 2025).

Ethanol production followed substrate consumption, reaching approximately 4.8% (v/v) at the end of the process, a value compatible with the typical range reported for meads (4%–12% v/v) and indicative of adequate fermentative conversion. The inverse relationship between sugars and ethanol confirms the efficiency of the fermentative metabolism of yeasts from the genus Saccharomyces, whose performance is strongly influenced by must composition and operational conditions such as temperature, pH, and nutrient availability (Pires et al. 2014; Kawa‐Rygielska et al. 2019). Additionally, the relatively low system pH (∼2.6–2.7) may have contributed to metabolic restrictions and reduced fermentative activity throughout the process, since excessively acidic conditions affect membrane permeability, intracellular ionic balance, and enzymatic activity in yeasts (Fleet 2008).

Although the final ethanol concentration (∼4.8%, v/v) was lower than the maximum values frequently reported for traditional meads, the substrate‐to‐product conversion coefficient (*Y*_P/S_ = 0.46 g/g) remained close to the theoretical ethanol yield for alcoholic fermentation (0.51 g/g). This finding indicates that the fermentative pathway itself remained highly efficient and that the moderate alcohol content was mainly associated with incomplete sugar consumption rather than metabolic inefficiency. Therefore, nutritional limitations inherent to honey, rather than carbon conversion efficiency, appear to have been the primary factor restricting ethanol accumulation. Similar behavior has been described for honey fermentations conducted without intensive nitrogen supplementation, in which high conversion yields coexist with relatively low ethanol concentrations due to premature arrest of yeast activity (Pereira et al. 2015; Ramalhosa et al. 2011; De Gino Soares et al. 2025).

**FIGURE 2:**
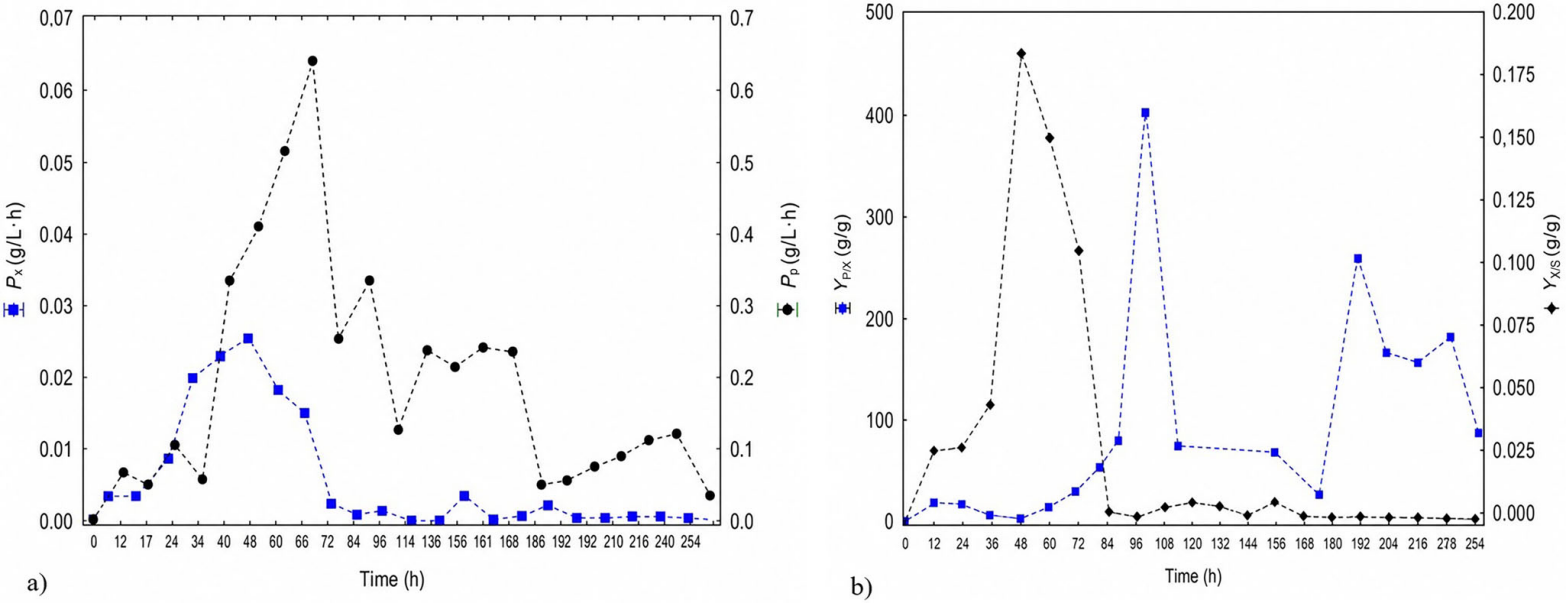
Kinetic profiles of mead fermentation during the fermentative process: (a) cell productivity (*P*_x_) and ethanol productivity (*P*_p_); (b) substrate‐to‐cell conversion factor (*Y*_X/S_) and substrate‐to‐product conversion factor (*Y*_P/S_) throughout the fermentation time.

The profiles observed in Figure 1 were corroborated by the kinetic parameters presented in Figure 2 and Table 2. The specific growth rate (*μ* = 0.028 h^−1^) and generation time (*t*_g_ = 24.92 h) were within the ranges reported for mead fermentations, which are characterized by relatively slow growth due to the natural nutrient limitations of honey (Li et al. 2026; Ramalhosa et al. 2011). These results also suggest that a significant portion of cellular metabolism was directed toward physiological maintenance and adaptation to fermentative stress rather than biomass synthesis.

**TABLE 2:** Kinetic and stoichiometric parameters of mead fermentation during exponential and total fermentation phases.

Parameters	Values	Unit	Growth phase
*μ*	0.028	h^−1^	Exponential
*t*_g_	24.92	h	Log
*P*_x_	0.017	g/L·h	Log
	0.003	g/L·h	Total
*P*_p_	0.192	g/L·h	Log
	0.163	g/L·h	Total
TRS	93.95	g/L	Total
*Y*_P/X_	10.96	g/g	Log
	50.96	g/g	Total
*Y*_X/S_	0.046	g/g	Log
	0.010	g/g	Total
*Y*_P/S_	0.510	g/g	Log
	0.460	g/g	Total
*Y*_p_ ^a^	47.90	g/L	Total
*Q*_S_ ^b^	0.376	g/L·h	Log
	0.319	g/L·h	Total

Abbreviation: TRS, total substrate consumption.

^a^ Volumetric product yield.

^b^ Volumetric substrate consumption rate.

Ethanol productivity (*P*_p_) showed similar values between the exponential phase (0.192 g/L·h) and the overall process (0.163 g/L·h) (Table 2), as did the substrate‐to‐product conversion factor (*Y*_P/S_), which varied from 0.510 to 0.460 g/g, indicating that substrate conversion into ethanol occurred relatively constantly throughout fermentation. This metabolic pattern suggests that ethanol production was partially uncoupled from cellular growth, a characteristic commonly observed in alcoholic fermentations conducted under nutritional limitation, in which fermentative metabolism remains active even after biomass stabilization (D'amore et al. 1989). This phenomenon is related to the fermentative metabolism characteristic of S. cerevisiae, an organism highly adapted to rapid sugar conversion into ethanol even under growth‐restricting conditions, a behavior associated with the Crabtree effect (Piskur et al. 2006).

On the other hand, cell productivity (*P*_x_) and the substrate‐to‐cell conversion factor (*Y*_X/S_) were significantly higher during the exponential phase, demonstrating that cellular growth was concentrated during this period. Cell productivity was approximately six times higher in the exponential phase compared to the overall process, whereas cellular yield was nearly five times higher during the same phase. The reduction in *P*_x_ and *Y*_X/S_ in the overall process evaluation results from the inclusion of lag and stationary phases, during which little or no biomass increase occurs. Under these conditions, the consumed substrate is predominantly directed toward metabolic maintenance and the production of secondary metabolites, especially ethanol.

During the lag phase, although no expressive biomass increase occurs, part of the substrate is consumed for metabolic adaptation, cellular maintenance, and synthesis of essential components required for the onset of exponential growth. In contrast, the stationary phase is characterized by biomass stabilization resulting from the equilibrium between viable and nonviable cells. At this stage, ethanol production may continue even after cellular growth cessation, a behavior widely described in alcoholic fermentations (Pires et al. 2014; Webster et al. 2025). Furthermore, the marked increase in the *Y*_P/X_ factor in the overall process evaluation (50.96 g/g), compared to the exponential phase (10.96 g/g) (Table 2), indicates partial uncoupling between cell growth and ethanol production, reinforcing that alcoholic synthesis remained active even after biomass stabilization. Collectively, the kinetic parameters indicate that growth limitation did not completely interrupt fermentative metabolism but redirected substrate consumption predominantly toward cellular maintenance and ethanol formation.

A limitation of the present study is that YAN and micronutrient concentrations were not determined. Although the fermentation profile strongly suggests nutritional limitations inherent to the honey must, direct measurement of these parameters would allow a more comprehensive assessment of their influence on fermentation kinetics, sugar utilization, and ethanol production. Future studies addressing these variables would contribute to a better understanding of the nutritional requirements of yeasts during mead fermentation and support the optimization of fermentation performance.

From a bioprocess perspective, the kinetic parameters indicate that fermentation became progressively maintenance‐associated after the exponential phase. The marked reduction in biomass productivity (*P*_x_), together with the sustained ethanol productivity (*P*_p_), demonstrates that carbon flux remained preferentially directed toward fermentative metabolism instead of cell proliferation. This metabolic shift is a well‐established physiological response of *S. cerevisiae* under nutrient depletion and ethanol stress, allowing ATP generation through glycolysis while minimizing the energetic cost of biomass synthesis (Gibson et al. 2007). Such behavior explains the persistence of ethanol production despite biomass stabilization and supports the interpretation that the fermentation was limited primarily by physiological stress rather than substrate availability.

### 3.3 Effects of Hibiscus Addition on the Properties of Mead

The physicochemical characterization of the hydroalcoholic H. sabdariffa extract (Table 3) demonstrated that it constituted a concentrated source of organic acids and antioxidant phytochemicals before its incorporation into the mead. The extract exhibited a markedly acidic pH (2.18), high titratable acidity (243.33 mEq/L), and elevated concentrations of total phenolic compounds (1.59 mg GAE/mL), flavonoids (0.091 mg QE/mL), and anthocyanins (0.441 mg CE/mL), as well as high antioxidant activity (2.69 mg TE/mL by ABTS and IC_50_ of 0.002 mL/mL by DPPH^•^). These compositional characteristics explain the physicochemical, chromatic, and functional modifications observed after incorporation of 20% (v/v) of the extract into formulation F2. Because the extract was incorporated only after alcoholic fermentation, its effects were restricted to post‐fermentation enrichment, improving acidity, color, phytochemical composition, and antioxidant activity without altering ethanol production or residual sugar levels.

**TABLE 3:** Physicochemical and color parameters of hibiscus extract, control mead, and hibiscus‐added mead.

Determinations	Hibiscus extract	Mead	
		F1 (control)	F2 (with hibiscus)
pH	2.18 ± 0.01	2.7^a^ ± 0.07	2.6^a^ ± 0.08
Total acidity (mEq/L)	243.33 ± 1.15	33.44^b^ ± 1.03	59.51^a^ ± 2.9
Alcohol content (%, v/v)	4.72 ± 0.15	4.78^a^ ± 0.18	4.69^a^ ± 0.22
Residual sugar (g/L)	—	37.02^a^ ± 1.51	36.74^a^ ± 1.23
HMF (mg/L)	—	19.00^a^ ± 0.88	18.90^a^ ± 0.65
Color parameters			
L*	15.23 ± 0.04	51.39^a^ ± 2.05	38.71^b^ ± 1.16
a*	11.51 ± 0.29	−3.16^b^ ± 0.09	6.4^a^ ± 0.19
b*	6.73 ± 0.16	11.59^a^ ± 0.35	6.2^b^ ± 0.24
Chroma C*	13.33 ± 0.33	12.01^a^ ± 0.36	8.9^b^ ± 0.27
Total anthocyanins (mg CE/mL)	0.441 ± 0.02	—	0.088^a^ ± 0.004
Total flavonoids (mg QE/mL)	0.091 ± 0.006	0.049^b^ ± 0.001	0.068^a^ ± 0.001
Total phenolic content (mg GAE/mL)	1.59 ± 0.07	0.132^b^ ± 0.004	0.448^a^ ± 0.014
Antioxidant activity			
ABTS^•+^ (mg TE/mL)	2.69 ± 0.10	0.016^b^ ± 0.009	0.176^a^ ± 0.015
DPPH^•^ (IC_50_—mL/mL)	0.002 ± 0.001	0.326^a^ ± 0.009	0.031^b^ ± 0.001

*Note*: Data represent mean values (*n* = 3) ± standard deviation. Equal letters within the same row indicate no significant difference at the 5% level (Student's *t*‐test) for the mead.

The reduction in pH and the significant increase in titratable acidity observed in hibiscus mead (F2) can be directly associated with the incorporation of organic acids naturally present in H. sabdariffa, such as citric, malic, and hibiscus acids, which are widely reported in the literature (Izquierdo‐Vega et al. 2020; Da‐Costa‐Rocha et al. 2014). In addition, hibiscus contains high concentrations of phenolic compounds and organic acids, which contribute not only to increased acidity but also to the microbiological stability of the beverage. Lower pH values may also contribute to anthocyanin stabilization, since acidic environments favor the flavylium cation form responsible for the characteristic red coloration of hibiscus pigments (Srinivasan and Rana 2025).

Although titratable acidity provides an overall measure of the acid content of the beverage, it does not distinguish individual organic acids. Therefore, the specific contribution of hibiscus‐derived organic acids, such as citric, malic, hibiscus, and other minor acids, could not be individually assessed in the present study. Future investigations should characterize the organic acid profile by high‐performance liquid chromatography (HPLC), allowing a more comprehensive evaluation of the relationship between acid composition, antioxidant activity, sensory attributes, and physicochemical stability of hibiscus‐enriched mead.

The phytochemical composition of the hydroalcoholic extract was directly reflected in the composition of the enriched mead. Total phenolic compounds increased from 0.132 to 0.448 mg GAE/mL, corresponding to an approximately 3.4‐fold increase, whereas total flavonoids increased from 0.049 to 0.068 mg QE/mL (approximately 39%). Anthocyanins, which were absent in the control formulation, reached 0.088 mg CE/mL in formulation F2. Considering that the extract contained 0.441 mg CE/mL of anthocyanins, these results indicate efficient transfer and retention of these pigments after incorporation. Anthocyanins, predominantly delphinidin‐3‐sambubioside and cyanidin‐3‐sambubioside, together with flavonoids and phenolic acids, are recognized as the major contributors to the antioxidant and chromatic properties of H. sabdariffa, acting through hydrogen atom transfer (HAT), electron donation, and metal‐chelating mechanisms (Da‐Costa‐Rocha et al. 2014; Khoo et al. 2017; Izquierdo‐Vega et al. 2020; Ojulari et al. 2019). Furthermore, interactions between anthocyanins and phenolic compounds naturally present in honey may promote copigmentation, contributing to greater color stability and visual intensity during storage and processing (Khoo et al. 2017; Da‐Costa‐Rocha et al. 2014).

Although the hibiscus extract was clarified using Whatman No. 1 filter paper prior to incorporation into the mead, this filtration step was intended solely to remove coarse insoluble particles derived from the floral material, thereby improving extract homogeneity and preventing the incorporation of suspended solids into the beverage. While some loss of particulate‐bound phenolic compounds or anthocyanins cannot be completely ruled out, the substantial increases observed in total phenolic compounds, flavonoids, anthocyanins, and antioxidant activity, along with the characteristic red coloration of formulation F2, indicate that the soluble bioactive compounds responsible for these effects were largely preserved after filtration. Therefore, the clarification procedure likely contributed to extract standardization without compromising the transfer of functional phytochemicals or the physicochemical and functional quality of the final mead.

**FIGURE 3:**
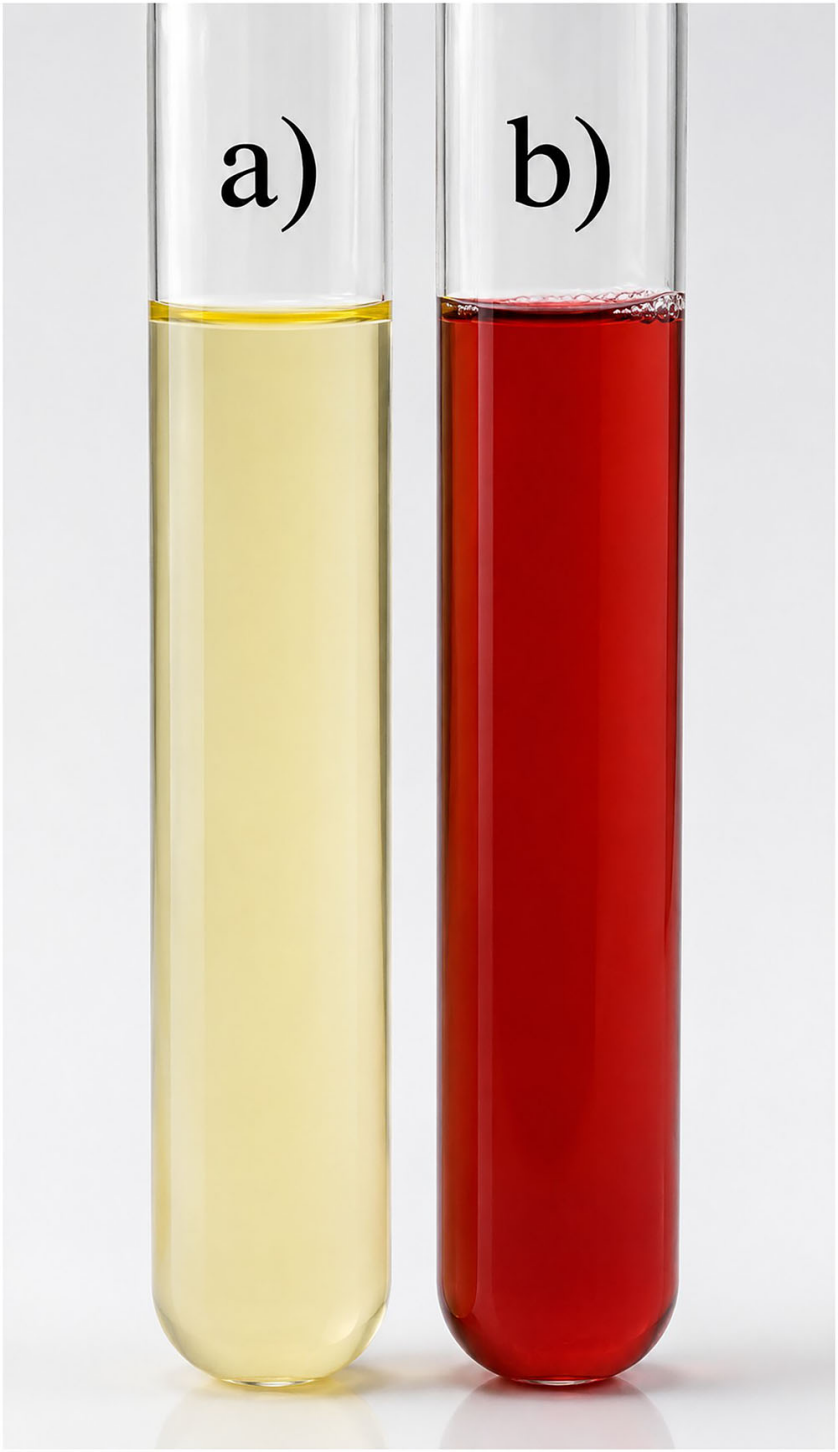
Visual appearance of mead formulations: F1—control (a) and F2—20% hibiscus extract (b).

Regarding color, perceptible changes were observed, demonstrating the strong contribution of hibiscus to the final appearance of the beverage. The control mead presented higher luminosity (L* = 51.39) and a negative a* value (−3.16), indicating lighter and slightly greenish tones. In contrast, hibiscus mead showed lower luminosity (L* = 38.71) and a positive a* value (6.4), revealing the presence of reddish hues characteristic of hibiscus natural pigments, especially anthocyanins. The b* parameter was also reduced (11.59 to 6.2), indicating lower contribution of yellowish tones, while chroma C* decreased from 12.01 to 8.9, suggesting a darker and less saturated color profile. The visual differences between formulations (Figure 3) corroborate the instrumental color measurements, demonstrating the strong impact of hibiscus pigments on the appearance of the beverage. Similar effects have been reported for fermented beverages enriched with phenolic‐rich botanical extracts, in which anthocyanin incorporation significantly improved color intensity and visual appeal (Cicha‐Wojciechowicz et al. 2025).

The enrichment of mead with hibiscus phytochemicals also resulted in a pronounced enhancement of antioxidant capacity. The ABTS assay increased from 0.016 to 0.176 mg TE/mL, corresponding to an approximately 11‐fold increase, whereas the DPPH assay showed a marked reduction in IC_50_ values from 0.326 to 0.031 mL/mL, indicating substantially greater radical scavenging efficiency. The excellent agreement between the ABTS and DPPH assays demonstrates that the antioxidant improvement was consistently detected using complementary analytical methods based on different reaction mechanisms. This enhanced antioxidant capacity is directly associated with the elevated concentrations of phenolic compounds, flavonoids, and anthocyanins transferred from the hydroalcoholic extract to the beverage. Moreover, synergistic interactions between hibiscus‐derived phytochemicals naturally present in honey may have further enhanced the radical scavenging capacity of the final product, as also reported by Essiedu and Kovaleva (2024).

From a chemical perspective, the marked increase in antioxidant activity cannot be attributed solely to the higher concentration of phenolic compounds introduced by the hibiscus extract but also to the complementary antioxidant mechanisms operating within the enriched mead matrix. Anthocyanins, flavonoids, and phenolic acids present in *H. sabdariffa* are capable of scavenging free radicals through HAT and single‐electron transfer (SET) mechanisms, while their ortho‐dihydroxylated aromatic structures also confer metal‐chelating capacity, reducing the formation of reactive oxygen species through Fenton‐type reactions. Furthermore, the acidic environment of mead favors the stabilization of anthocyanins in the flavylium cation form, preserving both their chromatic and antioxidant properties. Interactions between hibiscus anthocyanins and endogenous honey phenolics may additionally promote copigmentation phenomena, increasing molecular stability and prolonging antioxidant effectiveness. Together, these complementary mechanisms explain the substantial enhancement in antioxidant capacity observed after hibiscus incorporation, beyond a simple additive increase in total phenolic concentration (Khoo et al. 2017; Da‐Costa‐Rocha et al. 2014; Izquierdo‐Vega et al. 2020).

The similar alcohol and residual sugar concentrations observed between the two formulations confirm that hibiscus acted exclusively as a functional ingredient, enhancing the beverage's physicochemical, chromatic, phytochemical, and antioxidant properties while preserving the fermentation profile established during alcoholic fermentation. Consequently, both formulations were classified as mild meads under Brazilian regulations (Brazil 2012), based on a residual sugar content of approximately 37 g/L.

Overall, the results clearly demonstrate that the phytochemical composition of the hydroalcoholic *H. sabdariffa* extract directly determined the physicochemical and functional improvements observed in the enriched mead. Post‐fermentation enrichment efficiently transferred phenolic compounds, flavonoids, and anthocyanins to the beverage, simultaneously increasing antioxidant capacity, enhancing red coloration, and improving acidity. Therefore, the incorporation of *H. sabdariffa* after fermentation represents an efficient technological strategy for producing naturally colored, antioxidant‐rich, clean‐label meads with enhanced functional and commercial value.

These findings also indicate that post‐fermentation incorporation preserves thermolabile antioxidant compounds that might otherwise undergo degradation during active fermentation. Consequently, the enrichment strategy not only increases the concentration of bioactive molecules but also maximizes their chemical stability and biological functionality in the final beverage.

**FIGURE 4:**
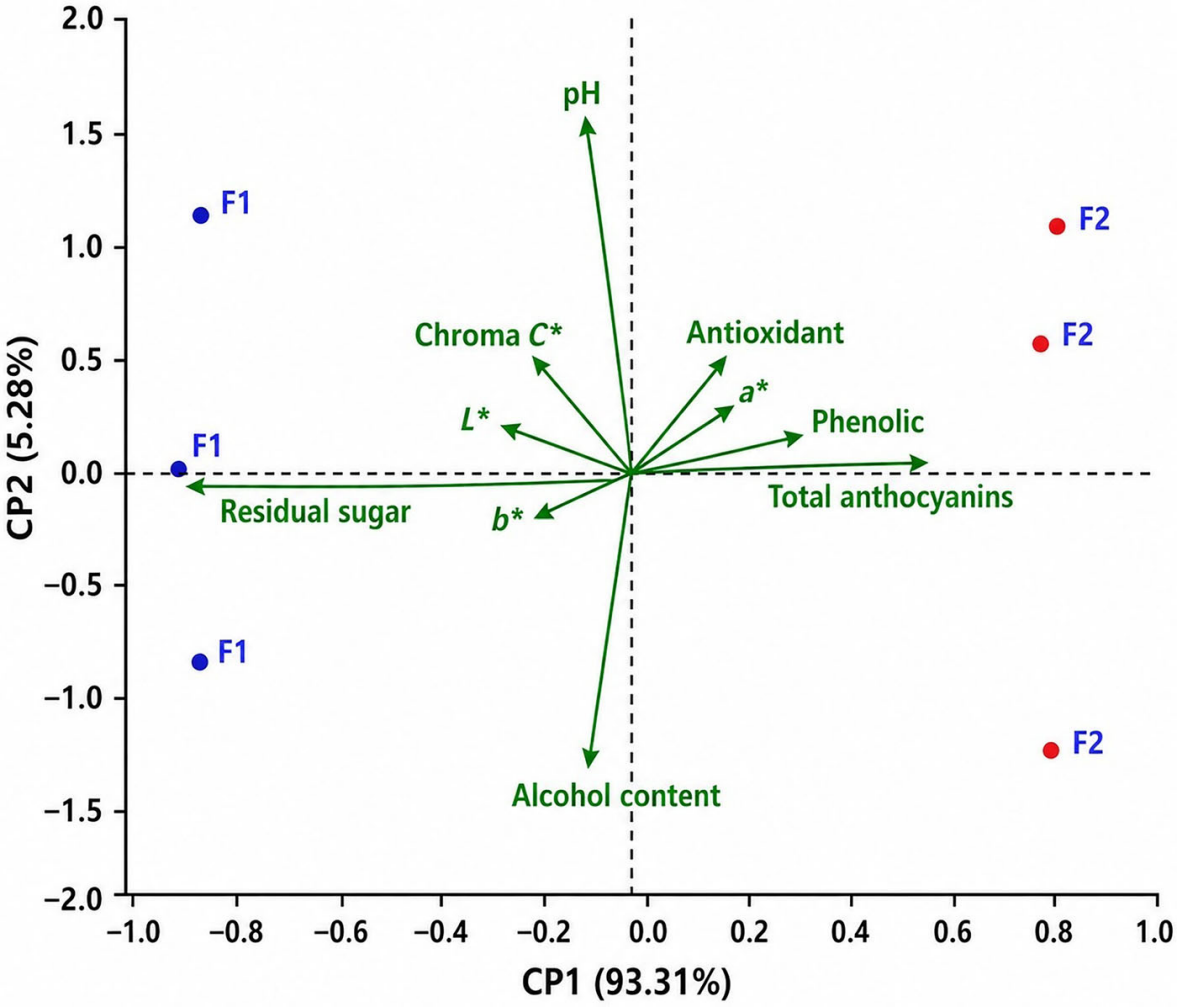
Principal component analysis (PCA) of physicochemical and color parameters of control mead (F1) and hibiscus‐added mead (F2).

To further explore the relationships among the physicochemical, chromatic, and antioxidant variables measured in the two experimental formulations, Pearson correlation analysis and principal component analysis (PCA) were performed (Table S1; Figure 4). These multivariate analyses were used as complementary exploratory tools to facilitate data visualization and identify patterns among the measured variables. The Pearson correlation matrix (Table S1) revealed strong associations among acidity, pH, color parameters, residual sugar, alcohol content, antioxidant activity, total phenolic content, total flavonoids, and total anthocyanins, highlighting the integrated behavior of these variables in the fermented beverage system. Total acidity exhibited a strong negative correlation with pH (r = −0.829), luminosity (L*) (r = −0.999), b* (*r* = −0.999), chroma C* (*r* = −0.999), and residual sugar (*r* = −0.967), while showing almost perfect positive correlations with the a* coordinate (*r* = 0.999), anthocyanins (*r* = 0.998), total phenolic compounds (*r* = 0.998), flavonoids (*r* = 0.996), and antioxidant activity (*r* = 0.966). These relationships indicate that the acidification promoted by hibiscus was intrinsically associated with the transfer of phenolic compounds and anthocyanins to the beverage, resulting simultaneously in enhanced antioxidant capacity and the development of the characteristic red coloration.

The exceptionally high positive correlations among anthocyanins, total phenolic compounds, flavonoids, and the a parameter (r = 0.992–0.999), together with their strong negative correlations with L*, b*, and chroma C* (*r* = −0.995 to −0.999), demonstrate that the chromatic changes observed in formulation F2 were directly driven by the accumulation of hibiscus‐derived phytochemicals. This behavior is characteristic of anthocyanin‐rich systems, in which the increasing concentration of red pigments reduces luminosity and yellow color intensity while shifting the CIELAB coordinates toward positive a* values. Moreover, the positive correlation between antioxidant activity and anthocyanins (*r* = 0.970), phenolic compounds (*r* = 0.967), and flavonoids (*r* = 0.944) confirms that these phytochemicals were the principal contributors to the enhanced radical scavenging capacity of the enriched mead.

The PCA (Figure 4) corroborated the correlation analysis by explaining 98.59% of the total data variability, with PC1 accounting for 93.31% and PC2 for 5.28%. The score plot showed a complete separation between formulations F1 and F2 along PC1, demonstrating that hibiscus incorporation was the predominant source of variation among the samples. Formulation F2 was located on the positive side of PC1, closely associated with anthocyanins, total phenolic compounds, flavonoids, antioxidant activity, total acidity, and the a* parameter, confirming that these variables jointly characterized the hibiscus‐enriched mead. Conversely, formulation F1 was positioned on the negative side of PC1, where pH, L*, b*, chroma C*, and residual sugar were the predominant variables, reflecting the lighter appearance and lower phytochemical content of the control beverage.

The loading plot further demonstrated that antioxidant activity, total phenolic compounds, flavonoids, anthocyanins, total acidity, and the a coordinate were oriented in the same direction, indicating a strong positive association among these variables. In contrast, pH, L*, b*, and chroma C* were positioned in the opposite direction, confirming their inverse relationship with the phytochemical and antioxidant characteristics of the hibiscus‐enriched mead. This distribution reinforces that the increase in acidity favored the stabilization of anthocyanins in their flavylium cation form, thereby simultaneously enhancing red coloration and antioxidant capacity.

Overall, the combined interpretation of the Pearson correlation matrix and PCA demonstrates that the incorporation of *H. sabdariffa* promoted integrated modifications in the physicochemical, chromatic, phytochemical, and functional properties of mead. Rather than acting independently, organic acids, anthocyanins, flavonoids, and phenolic compounds acted synergistically, producing a beverage with greater antioxidant potential, intensified red coloration, lower pH, and higher acidity while preserving its fermentative characteristics. These findings reinforce the technological potential of post‐fermentation hibiscus incorporation as an effective strategy for producing naturally colored, antioxidant‐rich meads with enhanced functional quality.

Because only two formulations were evaluated in the present study, the PCA and Pearson correlation analyses should be interpreted as exploratory rather than predictive. Future studies including multiple hibiscus concentrations will enable more robust multivariate analyses and a more comprehensive assessment of the relationships among physicochemical, color, and bioactive properties.

## 4 Conclusion

The incorporation of *H. sabdariffa* extract demonstrated that botanical ingredients can be successfully integrated into mead production to simultaneously enhance technological and functional quality. Post‐fermentation enrichment efficiently transferred phenolic compounds, flavonoids, and anthocyanins from the hydroalcoholic hibiscus extract to the beverage, resulting in increased total phenolic content, flavonoid concentration, anthocyanin content, antioxidant activity, and red color intensity, while preserving ethanol production and residual sugar levels.

The correlation analysis and PCA further demonstrated that these phytochemicals acted synergistically with the organic acids naturally present in hibiscus, promoting integrated improvements in acidity, chromatic attributes, and antioxidant capacity. The present study provides new evidence that hibiscus is not only a source of natural pigments and bioactive compounds but also an effective multifunctional ingredient capable of modulating key quality attributes of fermented beverages through the synergistic interaction of anthocyanins, phenolic compounds, and organic acids.

The incorporation of *H. sabdariffa* demonstrated that botanical ingredients can be successfully integrated into mead production to simultaneously enhance antioxidant activity through the combined action of anthocyanins, flavonoids, and phenolic acids under the acidic conditions of the mead matrix, while also improving the visual appeal of the beverage. Furthermore, the present work demonstrates the feasibility of using hibiscus as a natural alternative to synthetic colorants and functional additives, contributing to the development of clean‐label fermented beverages naturally enriched with bioactive phytochemicals. These findings also provide new insights into the relationship between phytochemical composition and the physicochemical, chromatic, and functional characteristics of hibiscus‐enriched mead.

Future research should investigate the sensory acceptance and consumer perception of hibiscus‐enriched mead, the stability and bioavailability of phenolic compounds during storage, and the influence of different hibiscus incorporation levels on the physicochemical, sensory, and functional properties of the beverage. Moreover, determining YAN and the micronutrient composition of honey must would provide valuable insights into the nutritional factors governing fermentation kinetics and efficiency. Finally, studies addressing fermentation optimization, volatile aroma composition, shelf life, and industrial‐scale production are warranted to facilitate the commercial development and broader application of hibiscus as a functional ingredient in fermented beverages.

## Author Contributions

**Luiz Antônio Teixeira Pedott**: conceptualization, investigation, visualization. **Lucas Henrique do Nascimento**: conceptualization, investigation, visualization. **Laís Thomazoni**: conceptualization, investigation, validation. **Jamile Zeni**: conceptualization, supervision, project administration, writing – review and editing. **Eunice Valduga**: conceptualization, supervision, writing – review and editing.

## Funding

This work was financially funded by the Coordination for the Improvement of Higher Education Personnel (CAPES)—Finance Code ROR 00x0ma614.

## Conflicts of Interest

The authors declare no conflicts of interest.

## Supporting information




**Supplementary Table**: jfds71439‐sup‐0001‐TableS1.docx
