# Supplementary material for: Impact of Hibiscus sabdariffa Incorporation on Physicochemical Properties, Color Attributes, and Antioxidant Activity of Mead
Source: J Food Sci. 2026 Sep 4;91(9):e71439. doi: 10.1111/1750-3841.71439 (PMC13544455; doi:10.1111/1750-3841.71439)
Supplement: Supplementary file 1 [file JFDS-91-0-s001.docx]

| **Variables** | **pH** | **Acidity** | **Alcohol** | **Sugar** | ***L**** | ***a**** | ***b**** | ***C**** | **AA** | **Anthocyanins** | **Flavonoid** | **Phenolic** |
| --- | --- | --- | --- | --- | --- | --- | --- | --- | --- | --- | --- | --- |
| **pH** | 1 |  |  |  |  |  |  |  |  |  |  |  |
| **Acidity** | **-0,829** | 1 |  |  |  |  |  |  |  |  |  |  |
| **Alcohol** | 0,380 | **-0,805** | 1 |  |  |  |  |  |  |  |  |  |
| **Sugar** | **0,707** | **-0,967** | **0,812** | 1 |  |  |  |  |  |  |  |  |
| ***L**** | **0,821** | **-0,999** | **0,814** | **0,969** | 1 |  |  |  |  |  |  |  |
| ***a**** | **-0,812** | **0,999** | **-0,819** | **-0,972** | **-0,999** | 1 |  |  |  |  |  |  |
| ***b**** | **0,836** | **-0,999** | **0,802** | **0,962** | **0,999** | **-0,998** | 1 |  |  |  |  |  |
| ***C**** | **0,841** | **-0,999** | **0,793** | **0,964** | **0,999** | **-0,999** | **0,999** | 1 |  |  |  |  |
| **AA** | **-0,777** | **0,966** | **-0,812** | **-0,928** | **-0,964** | **0,966** | **-0,966** | **-0,963** | 1 |  |  |  |
| **Anthocyanins** | **-0,823** | **0,998** | **-0,822** | **-0,960** | **-0,999** | **0,998** | **-0,999** | **-0,998** | **0,970** | 1 |  |  |
| **Flavonoid** | **-0,814** | **0,996** | **-0,807** | **-0,971** | **-0,997** | **0,996** | **-0,995** | **-0,996** | **0,944** | **0,992** | 1 |  |
| **Phenolic** | **-0,821** | **0,998** | **-0,825** | **-0,959** | **-0,999** | **0,998** | **-0,999** | **-0,998** | **0,967** | **0,999** | **0,993** | 1 |

**Table S1** Pearson correlation matrix (r) of physicochemical and color parameters of control mead and hibiscus-added mead

Note: Bold values indicate significant differences at p < 0.05 (r > 0.70).
